# Spontaneous Superficial Femoral Artery Pseudoaneurysm in Behcet's Disease

**DOI:** 10.1155/2014/860243

**Published:** 2014-04-16

**Authors:** Murat Ugurlucan, Selin Sendil, Omer Ali Sayin, Mehmet Barburoglu, Emre Gok, Gulsum Turkyilmaz, Murat Basaran, Ufuk Alpagut, Enver Dayioglu

**Affiliations:** ^1^Department of Cardiovascular Surgery, Istanbul Medical Faculty, Istanbul University, Millet Caddesi, Capa, Fatih, Istanbul, Turkey; ^2^Department of Radiology, Istanbul Medical Faculty, Istanbul University, Millet Caddesi, Capa, Fatih, Istanbul, Turkey

## Abstract

Behcet's disease is an autoimmune multisystemic disorder on vasculitis base. Cardiovascular involvement is the most important predictor of morbidity and mortality. The treatment should be planned carefully for pathologies requiring interventions. In our report, we present a 45-year-old patient with spontaneous superficial femoral artery pseudoaneurysm, our treatment strategy, and circumstances we faced.

## 1. Introduction

Behcet's disease is an autoimmune, multisystemic vasculitis. Its features were first described by Hulusi Behcet in 1937. The disease's hallmarks are recurrent oral aphthous ulcers, genital ulcers, and uveitis [1]. The incidence of the disease is similar between males and females. Behcet's disease is more common in Mediterranean and far-east Asian countries when compared to the other regions of the world. Behcet's disease can affect musculoskeletal system, the skin, gastrointestinal system, central nervous system, lungs, and cardiovascular system. HLA-B5 and HLA-B51 are proven to be associated with the disease and the detection of these antibodies is considered to be associated with bad prognosis [2–7].

In the course of the disease, while superficial venous pathologies such as thrombophlebitis or varicose veins are frequently seen, it is possible to face problems such as deep venous insufficiency, deep venous thrombosis, or even cava vein thrombosis. Arterial involvement is rare when compared to the venous involvement. Arterial involvement may emerge as arterial thrombosis, aneurysm, and pseudoaneurysm formations. Although rare, the cardiovascular involvement is the main predictor of the mortality and morbidity [2–5].

In our report, we present a patient who referred to our clinic with the diagnosis of spontaneous superficial femoral artery pseudoaneurysm.

## 2. Case Report

**Figure 1:**
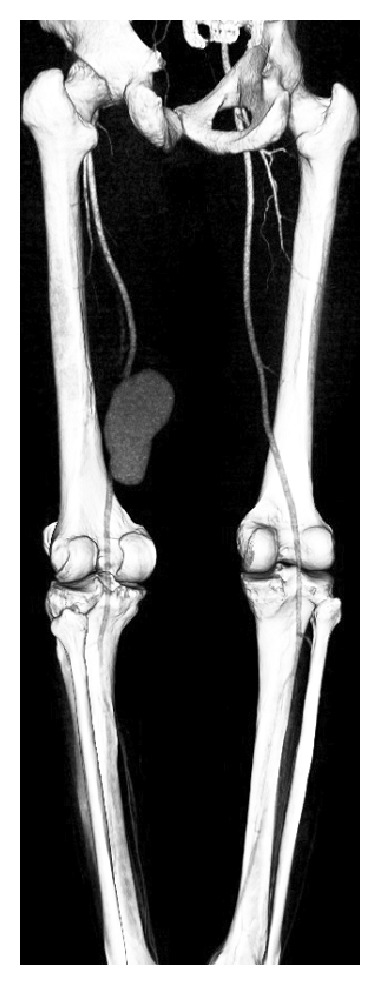
Computerized tomography angiography indicated giant pseudoaneurysm of the superficial femoral artery (posterior view).

A 45-year-old male patient was referred to our clinic with the diagnosis of left superficial femoral artery pseudoaneurysm detected by Doppler ultrasonography that was performed to investigate the pulsating mass in the left upper thigh and edema of the particular extremity. He had been diagnosed with Behcet's disease three years ago with the symptoms of recurrent oral aphthous ulcers, bilateral lower extremity repeating venous thrombosis, and positive Pathergy test. In his history, there were left inguinal hernia operation in 1994, left meniscus operations in 2003 and 2007, and gastric bleeding in 2001 and 2003. Familial Mediterranean fever was diagnosed in his uncle and cousin, coronary artery disease in his sister, and valvular heart disease in another sister as family history. He received pulse steroid therapy (1 gr methylprednisolone) for 3 days followed by 60 mg methylprednisolone and 1 gr cyclophosphamide per day as soon as the pseudoaneurysm was detected. His symptoms did not relieve and the pulsating mass enlarged despite immunosuppressive therapy. The arterial pathology was confirmed with computerized tomography angiography, which revealed an 117 × 63 × 75 mm pseudoaneurysm confined to the mid segment of the superficial femoral artery, which compressed and occluded the superficial femoral vein (Figure 1). We decided surgical treatment after consulting the patient with the rheumatology clinic and following the consent of him.

**Figure 2:**
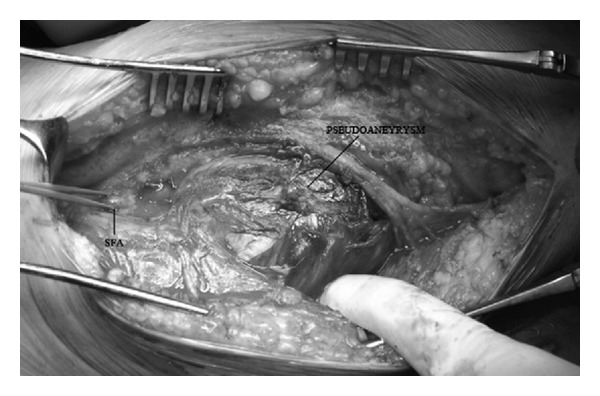
Severely destructed superficial femoral artery and the pseudoaneurysm (*SFA, *superficial femoral artery).

**Figure 3:**
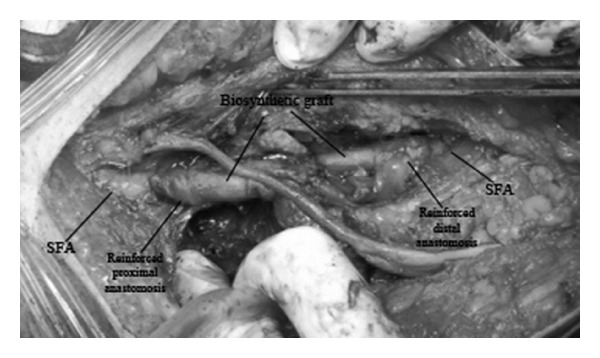
Excision of the destructed segment of the superficial femoral artery and biosynthetic graft interposition with anastomosis sites reinforcements (*SFA, *superficial femoral artery).

Operation was performed with general anesthesia. The pseudoaneurysm was approached through a direct incision above the artery at the mid segment of the thigh. The relatively disease-free segments of the proximal and distal superficial femoral artery were looped and controlled. The diseased segment of the artery was severely destructed (Figure 2) and a repair was not suitable. Following heparinization the diseased segment of the artery was replaced with biosynthetic vascular graft interposition (Omniflow II, 6 mm × 60 cm, Bionova). The anastomosis regions were augmented with graft materials rolled around the anastomosis to prevent pseudoaneurysm formation (Figure 3). Postoperative course was complicated with surgical site infection and reexploration. The graft was excised and saphenous vein, which was harvested from the contralateral leg, was interposed between the relatively healthy segments of the superficial femoral artery for the treatment. Again the anastomoses were reinforced with segments of saphenous vein. The culture of the extracted specimen indicated* E. coli* and the patient was treated with ciprofloxacin. On the third postoperative day, the patient was again taken to the operating theatre due to severe bleeding. The saphenous vein was found to be destructed and replaced with a biosynthetic graft. Immunosuppressive regime of the patient was not stopped during his hospitalization period or interventions and reinforced with 1 gr methylprednisolone before and after the operations. He was discharged home on the seventh postoperative day despite the fact that postoperative course was complicated with elongated serous discharge from the incision which stopped after 42 days. He had been free of symptoms, received immunosuppressive therapy, and followed up regularly.

The histopathologic examination of the excised arterial segment revealed focal foamy macrophage accumulation, marked fibrosis at the intima, and chronic nonspecific inflammation at the adventitia level of the vessel.

## 3. Discussion

Behcet's disease is an autoimmune multisystemic syndrome without an identified etiology in the current era. Symptoms occur secondary to vasculitis and the complaints of the patients tend to recur [2–5]. There is no certain diagnostic laboratory test and the patient's complaints raise a clinical suspicion.

Behcet's disease is particularly seen between the second and fourth decades of life; however, in the same age group, the course of the disease is worse in males. Except the classic triad, the superficial venous thromboses are common [3–5] and may be the predictors of more serious future cardiovascular problems. In the acute setting, heparin derivatives as the classic venous thrombosis treatment are beneficial; however, since the main pathology depends on the autoimmune vasculitis, despite the heparin therapy, the thrombosis tends to progress and deep vein thrombosis, caval vein thrombosis may even lead to further cardiac and other complications. It is mandatory to suppress the immune system and immunosuppressive agents should be added to the treatment [2–7]. Arterial involvement is not very rare in undiagnosed patients or in patients with insufficient immunosuppression. Stenosis, thrombosis, and true or false aneurysms can occur. In patients with Behcet's disease the main determinant of mortality and morbidity is the cardiovascular involvement [2–5].

When the vascular specimen obtained from patients with Behcet's disease for any reasons is examined under the light microscope, it reveals thickening of the adventitia, elastic and muscular fiber loss in the media layer, and fibrosis of the intima [4]. As the natural result of these microscopic findings in Behcet's disease, stenosis of the small vessels and the true or false aneurysms of the large vessels are common. These histopathologic findings also explain why vascular interventions in patients with Behcet's disease may be eventful. After the percutaneous or surgical interventions restenosis, pseudoaneurysms, or aneurysms can frequently appear in the inflamed vascular structures. For the vascular lesions, if the intervention is needed and surgery is inevitable, primary repair of the affected segment should preferably be attempted and when it is not possible, replacement should be performed. If a graft is going to be used it should be anastomosed to the disease-free segments of the vessel. Otherwise pseudoaneurysm risk is high at the anastomosis line [2–5]. In our patient we preferred an artificial graft material during his first operation; however, needed to replace the vessel due to infection with a saphenous vein segment. The vein was harvested from the contralateral leg, as there was leg edema at the affected side.

Another factor, which affects the success and complication rate of the surgery in patients with Behcet's disease, is the timing (acute flare-up period or the remission status of the disease) of the planned intervention. If no emergent intervention is needed, the procedures should be performed during the remission period when the vasculature is more stable, not in an acute inflammation period. In situations, which an emergent procedure is inevitable, high dose steroids should be administered before the intervention and continued after. Such strategy may decrease the complication rate [2–5]. Our patient had a complicated huge superficial femoral artery pseudoaneurysm which compressed the superficial femoral vein and lead to leg edema. Rather than a radiologic interventional treatment, we preferred conventional open surgery both to treat the aneurysm and relieve venous compression. Literature includes reports of endovascular treatment of vascular pathologies in patients with Behcet's disease with variable promising outcomes [8, 9].

In conclusion, Behcet's disease is an autoimmune multisystemic vasculitis. The most important determinant of the prognosis in patients is the cardiovascular involvement. In order to control the symptoms immunosuppressive agents should be used. However, despite high dose of multiple immunosuppressives acute exacerbations and vascular pathologies can occur. When complications occur, medical therapy should be revised and taken into account in the first order. In unresolving and emergent cases, an interventional therapy may be required. Otherwise, for the interventions especially, the remission periods should be waited. Before and after an intervention high dose immunosuppressive agents should be used to decrease the complication rate.
